# Prevalence of Avoidant/Restrictive Food Intake Disorder in Portuguese children aged 2–10 years: a cross-sectional study

**DOI:** 10.1186/s40337-025-01456-y

**Published:** 2025-11-26

**Authors:** Rita Novo, Leonel Vieito, Sara Simões Dias, Cátia Braga-Pontes

**Affiliations:** 1 ciTechCare – Centre for Innovative Care and Health Technology, Polytechnic University of Leiria, Leiria, Portugal; 2 School of Health Sciences, Polytechnic University of Leiria, Leiria, Portugal

**Keywords:** Avoidant/Restrictive Food Intake Disorder, Prevalence, Children, Eating behaviour

## Abstract

**Background:**

Avoidant/Restrictive Food Intake Disorder (ARFID) is an eating disorder characterised by the avoidance or restriction of food intake, associated with high sensory sensitivity, disinterest in eating, or fear of adverse consequences. It can lead to nutritional deficiencies, impaired growth, or psychosocial difficulties. Although recognition of ARFID has increased internationally, prevalence data in Portugal remain scarce.

**Methods:**

This cross-sectional, descriptive observational study assessed the prevalence of ARFID in children aged 2–10 years attending a private outpatient clinic. Paediatricians applied DSM-5-TR diagnostic criteria during routine consultations. Parents completed online questionnaires, including the Children’s Eating Behaviour Questionnaire (CEBQ) and the Child Feeding Questionnaire (CFQ).

**Results:**

Of the 163 children assessed, ARFID was diagnosed in 3.1% of cases. Four of the five identified children were male (80%), with a mean age of 5.8 years (SD = 2.17). On the CEBQ, children with ARFID showed the highest mean scores on the food fussiness and satiety responsiveness subscales, indicating greater selectivity and heightened sensitivity to fullness. The lowest mean scores were observed in emotional overeating and desire to drink. On the CFQ, perceived responsibility and monitoring were the subscales with the highest mean values.

**Conclusions:**

This study provides the first prevalence estimate of ARFID in Portuguese clinical practice. Although the small sample limits generalisability, the findings emphasise the need to raise awareness among both clinicians and parents to promote earlier recognition, thereby improving support for children and families and helping to reduce the long-term impact of ARFID.

## Abstract (plain language summary)

This study examined how prevalent Avoidant/Restrictive Food Intake Disorder (ARFID) is among Portuguese children aged 2 to 10 years old. ARFID is an eating disorder in which a child avoids or restricts certain foods, which can lead to problems like poor growth, nutritional gaps, or social difficulties. Although awareness of ARFID is growing worldwide, research in Portugal is still minimal. In this study, paediatricians assessed 163 children during routine appointments, and parents completed questionnaires about their child’s eating habits and how they feed their child. The results showed that about 3 in every 100 children had ARFID, and most of these were boys around five or six years old. Children with ARFID were more sensitive to feelings of fullness and were fussier about what they ate. Parents of these children tended to monitor what their child eats more closely. By understanding how common ARFID is and how it relates to parenting, this study highlights the need for early detection, better guidance for families, and training for health professionals to support children with eating difficulties in their daily lives.

## Introduction

Avoidant/Restrictive Food Intake Disorder (ARFID) is defined as a persistent avoidance or restriction of food intake, resulting in significant weight loss, significant nutritional deficiency, dependence on enteral feeding or oral nutritional supplements, or marked interference with psychosocial functioning. ARFID may arise from sensory sensitivities, adverse eating-related experiences (e.g., choking, vomiting), or a lack of interest in food. It can also involve specific food aversions or fear of eating-related consequences. Unlike Anorexia Nervosa (AN) or Bulimia Nervosa (BN), ARFID occurs in the absence of body image concerns [1, 2]. It was introduced in the 5^TH^ Edition of the Diagnostic and Statistical Manual of Mental Disorders (DSM-5) in 2013, within the category of Feeding and Eating Disorders (FED), and is also classified under code 6B83 in the International Classification of Diseases 11th Revision [3].

Robust data on the incidence and prevalence of ARFID in the general population are still scarce. The 5^TH^ Edition of the Diagnostic and Statistical Manual of Mental Disorders, Text Revision (DSM-5-TR) estimated a frequency of 0.3% among individuals aged 15 years or older [2], although this is based on limited data. Recent epidemiological data from the United Kingdom (UK) and Ireland reported an annual incidence of ARFID of 2.79 per 100,00 children and adolescents (95% CI 2.48 to 3.13) [4]. Regarding prevalence, a 2022 systematic review reported prevalence rates of 5%−22.5% in specialised eating disorder services, rising to 32%–% in specialised feeding clinics. In non-clinical samples, rates ranged from 0.3% to 15.5% [5]. Retrospective studies using DSM-5 criteria identified a prevalence between 13.8% and 22.5%, notably higher in clinical contexts. In these studies, children and adolescents with ARFID were typically younger and predominantly male, compared to patients with AN, BN, or other eating disorders. They also more frequently exhibited fear of choking or vomiting, reliance on supplements, and higher rates of comorbid anxiety and neurodevelopmental disorders [6–8]. In a large paediatric sample, a French study found a 3% prevalence, with children diagnosed with ARFID being younger (mean age: 4.8 years) than those with other eating disorders [9]. Although no prevalence data are available for Portuguese children in public or private healthcare settings, a school‑based survey reported that 15.5% of primary‑schol children aged 5–10 years screened positive for ARFID symptoms using a DSM‑5‑derived questionnaire [10].

ARFID frequently co-occurs with neurodevelopmental conditions such as autism spectrum disorder (ASD), Attention-Deficit/Hyperactivity Disorder (ADHD), and anxiety. Affected individuals often present cognitive inflexibility, rigidity around mealtimes and food types, and strong routines – traits commonly linked to ASD. In one study, 50% of paediatric ARFID patients also had anxiety [11], while others have highlighted associations with ADHD [7].

In children and adolescents, such patterns are associated with health risks, including weight loss, nutritional deficiencies, and impaired growth and development [12]. ARFID commonly results in reduced intake of macronutrients (proteins, fats, carbohydrates) and energy, as well as deficiencies in micronutrients such as vitamins B1, B2, B9, B12, C, and K, and minerals like zinc, potassium, and iron [12, 13].

Parental influence is a key factor in children’s eating behaviours. Inadequate parenting models, irregular meal routines, and inappropriate feeding practices have been identified as risk factors for feeding difficulties [14, 15]. Children with feeding issues are less likely to participate in family meals, more likely to consume the same foods repeatedly, and often eat with distractions such as television [14]. Restrictive parental feeding practices, such as limiting access to specific palatable foods, are generally counterproductive, as they increase preference for restricted foods and contribute to weight gain, whereas moderate or authoritative approach, which combines clear limits with a supportive feeding environment, allows children autonomy over how much they eat and has been shown to promote self-regulation, improve dietary quality, and reduce feeding problems. Similarly, pressure to eat has been associated with food avoidance behaviours and reduced intake of fruits and vegetables [16, 17]. These dynamics may be particularly relevant for children with ASD, who not only show higher rates of ARFID, food neophobia, selective eating, and emotional under-eating compared to those without clinical conditions, but also tend to experience greater parental pressure to eat [18].

Understanding the interplay between ARFID, parental feeding practices, and children’s eating behaviours is essential to supporting children and their families, promoting healthier eating habits, and mitigating the long-term consequences of ARFID.

This study aims to assess the prevalence of ARFID in children aged 2–10 years in a clinical setting, characterise affected children, and explore associations with anthropometric, sociodemographic, parental and behavioural variables.

Based on previous evidence, we hypothesised that ARFID would occur more frequently on male children compared to female, and that affected children would more often present with comorbid AD and ASD.

## Methods

### Study design and participants

This descriptive cross-sectional observational study included children aged 2–10 years followed in an outpatient paediatric clinic in Portugal. The clinic provides routine paediatric care alongside access to different medical and therapeutic specialties in an ambulatory setting.

Caregivers who could not understand Portuguese were excluded.

The required sample size of 163 children was calculated based on an estimated ARFID prevalence of 13.3%, derived from the mean of four comparable studies [6, 8, 9, 19]. The calculation assumed a 95% confidence level, a 5% margin of error, and considered the clinic’s paediatric population of approximately 2,060 children.

The Ethics Committee of the Polytechnic Institute of Leiria approved the study on 20 October 2023 (CE/IPLEIRIA/55/2023).

### Procedure

Seven paediatricians evaluated the presence of ARFID during routine consultations between 13 November 2023 and 5 April 2024, applying DSM-5-TR diagnostic criteria.

Before data collection, they were provided with a concise summary of the DSM‑5‑TR diagnostic criteria for ARFID and additional guidance to ensure a common understanding. Participation was voluntary, and the paediatricians agreed to apply these criteria consistently during their routine consultations. These assessments were integrated into usual clinical practice and did not involve additional formal instruments.

Following the consultations, parents or caregivers completed online sociodemographic, clinical, and dietary questionnaires, with responses accepted until 24 April 2024.

Participation was voluntary, and informed consent was obtained before the consultation. All participants were informed of the study objectives and their right to withdraw at any time. Clarifications were provided as needed to ensure comprehension. Anonymity was maintained through the use of alphanumeric codes.

A QR code printed on the consent form provided direct access to the LimeSurvey questionnaires.

### Materials

#### Assessment of the presence of ARFID

Paediatricians assessed the presence of ARFID during consultations using the DSM-5-TR diagnostic criteria [1]. Cases were included if they met Criterion A, characterised by changes in eating behaviour – such as lack of interest in food, sensory-based avoidance, or fear of aversive consequences— accompanied by at least one of the following: significant weight loss (or failure to achieve expected weight gain or faltering growth in children)(A1), significant nutritional deficiency (A2), dependence on enteral feeding or oral nutritional supplements (A3), or marked interference with psychosocial functioning (A4).

Sex, age and anthropometric data (height in cm and weight in kg) were also collected.

#### Sociodemographic and clinical questionnaire

A sociodemographic and clinical questionnaire was developed to characterise the sample, comprising two sections for caregivers and the child.

The caregiver section collected data on age, marital status, education, household income, gestational history (e.g., maternal weight gain, type of delivery, pregnancy complications), and current health conditions.

The child section addressed feeding practices in the first year of life, including breastfeeding duration, complementary feeding, and current issues. Caregivers were specifically asked to report any current food aversions, allergies, or medical conditions.

#### Children’s Eating Behaviour Questionnaire (CEBQ)

Parents completed the Children’s Eating Behaviour Questionnaire (CEBQ), a validated and reliable tool developed to assess eating behaviours in children from two years of age [20].

The CEBQ includes 35 items rated on a 5-point Likert scale (“1 - Never” to “5 - Always”), with higher scores indicating more pronounced eating behaviours. These are grouped into two domains: *food avoidance* (Satiety Responsiveness (SR), Slowness in Eating (SE), Food Fussiness (FF), and Emotional Undereating (EUE)), and *food approach* (Food Responsiveness (FR), Enjoyment of Food (EF), Emotional Overeating (EOE), and Desire to Drink (DD)). The CEBQ has been used alongside ARFID screening instruments to describe eating patterns, supporting its relevance in this context [21].

The Portuguese version was validated in 240 children aged 3–13 years [22, 23].

#### Child Feeding Questionnaire (CFQ)

The Child Feeding Questionnaire (CFQ) is a self-report instrument designed for parents of children aged 2–11, assessing beliefs, attitudes, and practices related to child feeding [24]. The CFQ comprises 31 items across seven subscales. Four subscales assess *risk and concern about weight* (Perceived Responsibility (RESP), Parent Perceived Weight (PPW), Perceived Child Weight (PCW), and Parents’ Concerns about Child Weight (CN)), and three reflect *parental control attitudes* (Restriction (RST), Pressure to Eat (PE), and Monitoring (MN)). While it has not been widely applied in ARFID studies, it is a validated instrument for assessing parental feeding practices and was included to capture family influences on children’s eating behaviour. Items are rated on a 5-point Likert scale. The CFQ has been validated for the Portuguese population [25, 26].

### Statistical analysis

Data were analysed using IBM SPSS Statistics (v26).

Quantitative variables were described by means and standard deviations, and categorical variables by absolute and relative frequencies.

Internal consistency of the CEBQ and CFQ was assessed using Cronbach’s Alpha. A 5% significance level was assumed.

BMI-for-age and sex were calculated using WHO Anthro (v3.2.2) for ages 2–5 and WHO AnthroPlus software (v1.0.4) for children older than 5 years.

## Results

A total of 163 assessments were conducted during paediatric consultations. After excluding incomplete or duplicate responses, 94 complete questionnaires were analysed.

### Sample results

Of the 163 children, 54.4% were female, and the mean age was 5.42 years (SD = 2.48). Most (95.5%) had normal weight; six were overweight, and one was obese. Table 1 details sociodemographic and anthropometric data.

**Table 1:** Sociodemographic and anthropometric data of participating children

	*n*	%
Sex		
Female	86	54.4
Male	72	45.6
Nutritional status		
Normal weight	148	95.5
Overweight	6	3.9
Obesity	1	0.6

Most questionnaires were completed by mothers (94.7%), with five completed by fathers. The majority of caregivers were Portuguese (97.9%), had higher education (85.2%), and were married or in a common-law marriage (92.6%). Over half (54.3%) had two children, and 9.6% had three. Table 2 summarises caregivers’ characteristics.

**Table 2:** Sociodemographic characterisation of parents/caregivers

	*n*	%
Sex		
Female	89	94.7
Male	5	5.3
Nationality		
Portuguese	92	97.9
Brazilian	1	1.1
French	1	1.1
Educational qualifications		
2nd Cycle of Elementary School	1	1.1
Secondary education	13	13.8
Undergraduate Degree	59	62.8
Master’s Degree	20	21.3
Doctorate Degree	1	1.1
Monthly Income		
< 599€	1	1.2
600€ − 999€	4	4.7
1000€ − 1499€	5	5.9
1500€ − 1999€	20	23.5
2000€ − 2499€	23	27.1
> 2500€	32	37.6

### ARFID case results

Five children (3.1%) were diagnosed with ARFID, with a mean age of 5.80 years (SD = 2.17); four were male (80%), and one female (20%). All had adequate weight for age, though two exhibited significant weight loss (A1), and two had marked psychosocial impairment (A4), with one child meeting both criteria (A1 and A4). Among the ARFID cases, reported contributing factors were a history of choking, adverse medication effects, and co-occurrence with ASD.

#### Pregnancy, clinical, and dietary data

Among the ARFID cases (*n* = 5), pregnancy and parental clinical data were available for three children, as only three caregivers completed this section of the questionnaire. Of these, one child was born prematurely and another via Caesarean section. Maternal weight gain during pregnancy averaged 14.3 kg, and maternal conditions included one case of obesity and one of pre-eclampsia. Postnatal complications included one case of postpartum depression.

During data collection, maternal obesity and paternal hypertension were reported in one case each.

In terms of child clinical and dietary features, one case presented a caregiver-reported food aversion specifically to cauliflower. In addition, one caregiver reported that their child had ADHD; however, this was not confirmed during paediatric consultation. No further comorbidities or specific food aversions were reported.

#### Children’s Eating Behaviour Questionnaire (CEBQ) results

Eating behaviours were assessed using the CEBQ. Internal consistency, measured by Cronbach’s Alpha, ranged from 0.67 to 0.89 across the eight subscales and the total score.

Among ARFID cases, the mean total CEBQ score was 2.638 (SD = 0.368), with values ​​ranging from 2.23 to 2.94. The FF and SR subscales observed the highest mean scores and the lowest in EOE and DD.

*Food approach* behaviours (EF, FR, EOE, DD) had a mean of 1.954 (SD = 0.356), ranging from 1.67 to 2.35.

*Food avoidance* behaviours (SR, SE, FF, EUE) had a mean of 3.172 (SD = 0.457) ranging from 2.66 to 3.55.

Due to limited complete responses, associations with sex, age or Body Mass Index (BMI) could not be established.

Table 3 details the descriptive statistics for each subscale and the total score.

**Table 3:** CEBQ descriptive analysis in children diagnosed with ARFID (*n* = 3)

CEBQ subscales	Children’s eating behaviour		
	Min-max	Means ± SD	Median (Q1-Q3)
EF - Enjoyment of food	2.00–3.00	2.500 ± 0.500	2.500 (2.000-)
FR - Food responsiveness	1.60–3.00.60.00	2.067 ± 0.808	1.600 (1.600-)
SR - Satiety responsiveness	2.75–3.75	3.167 ± 0.520	3.000 (2.750-)
SE - Slowness in eating	2.40–3.60	3.122 ± 0.643	3.400 (2.400-)
FF - Food fussiness	3.00–3.83.00.83	3.389 ± 0.419	3.333 (3.000-)
EOE - Emotional overeating	1.25–1.75	1.583 ± 0.289	1.750 (1.250-)
EUE - Emotional undereating	2.50–3.50	3.000 ± 0.500	3.000 (2.500-)
DD – Desire to drink	1.33–2.00.33.00	1.666 ± 0.333	1.667 (1.333-)
Food approach (EF, FR, EOE, DD)	1.67–2.35	0.356 ± 1.67	1.838 (1.671-)
Food avoidance (SR, SE, FF, EUE)	2.66–3.55	3.172 ± 0.457	3.308 (2.663-)
Total CEBQ	2.23–2.94	2.638 ± 0.368	2.743 (2.229-)

#### Child Feeding Questionnaire (CFQ) results

Internal consistency of the seven CFQ subscales and total score, assessed via Cronbach’s Alpha, ranged from 0.66 to 0.94.

The highest mean values ​​were in the RESP and MN subscales, and the lowest in CN and PCW.

*Risk and concern about weight* domain (RESP, PPW, PCW, CN) had a mean of 3.67.

*Parental control attitudes* ranged from 2.61 to 3.71, with a mean of 3.282 (SD = 0.588). The overall CFQ mean score was 3.367.

Due to the small number of responses, associations with sex, age or BMI could not be assessed. Table 4 presents the descriptive statistics for each CFQ dimension.

**Table 4:** CFQ descriptive analysis of the children diagnosed with ARFID (*n* = 3)

CFQ subscales	Parental control over children’s food		
	Min-Max	Means ± SD	Median (Q1-Q3)
RESP - Perceived responsibility	3.67–5.00.67.00	4.222 ± 0.694	4.000 (3.667-)
PPW - Parent perceived weight	3.00–3.50.00.50	3.167 ± 0.389	3.000 (3.000-)
PCW - Perceived child weight	2.80–2.80	2.800	2.800 (2.800–2.800)
CN - Parents’ concerns about child weight	1.00–3.00	2.222 ± 1.072	2.667 (1.000-)
RST - Restriction	3.25–3.63	3.375 ± 0.217	3.250 (3.250-)
PE – Pressure to eat	1.25–4.00.25.00	2.917 ± 1.465	3.500 (1.250-)
MN - Monitoring	3.33–4.00.33.00	3.556 ± 0.385	3.333 (3.333-)
Concern about weight (RESP, PPW, PCW, CN)	3.37–3.37	3.367	3.367 (3.367–3.367)
Parental control attitudes (RST, PE, MN)	2.61–3.71	3.282 ± 0.588	3.528 (2.611-)
Total CFQ	3.37–3.37	3.367	3.367 (3.367–3.367)

## Discussion

This study identified an ARFID prevalence of 3.1% in children aged 2–10 years, based on 163 assessments in a private paediatric clinical setting. However, only 94 participants (57.7%) completed the questionnaire, reflecting limited engagement. The small number of ARFID cases limits the strength of the conclusions that can be drawn, and the low completion rate of questionnaires among these children further restricts the analysis. Nevertheless, these findings provide preliminary insight into the presence and characteristics of ARFID in Portuguese clinical settings.

The 3.1% prevalence of ARFID identified in children aged 2–10 years in this study is consistent with Bertrand et al. (2021), who reported a 3.0% prevalence in general paediatric consultations among children aged 0–18 years (mean age 4.8 years) [9]. By contrast, studies conducted in specialised eating disorder services have reported substantially higher rates, ranging from 13.8% to 22.5% [6, 19], reflecting the more severely affected populations typically seen in those settings. Complementary evidence from population-based surveillance was provided by Sánchez-Cerezo et al. (2024), who reported an annual incidence of 2.79 per 100,000 children and adolescents across the UK and Ireland, underscoring the growing recognition of ARFID in public health monitoring. Ultimately, a recent meta-analysis synthesizing 26 studies reported pooled prevalence estimates of 11.14% under random-effects models and 4.51% when accounting for study quality [27]. Although derived from heterogeneous populations across all ages and settings, these estimates underscore the considerable variability in reported ARFID prevalence and highlight the need for more methodologically robust research.

Regarding sex distribution, as hypothesised, 80% of ARFID cases in this study were male, in line with reports of a male predominance in ARFID diagnoses [5, 28].

The mean age of children diagnosed with ARFID was 5.80 years (SD = 2.17), and the observed prevalence of 3.1% supports previous research that indicates that ARFID is more commonly identified in early childhood than in adolescence [5, 28]. However, because the present study included only children aged 2–10 years, it was not possible to explore how prevalence varies across age groups. Further research involving broader age ranges is needed to clarify age-related differences in ARFID prevalence.

Children with ARFID often present with co-occurring conditions, most notably neurodevelopmental disorders (e.g., ASD), as well as a high prevalence of AD [5]. As hypothesised, ARFID cases were expected to show higher rates of comorbid anxiety and ASD. In our study, one case of AD and ADHD was reported by caregivers, and a paediatrician confirmed one case of ASD, corresponding to 20% of the ARFID cases in each instance. Nicely et al.. (2014) and Fisher et al. (2014) reported AD comorbidity rates of 19.4% and 72%, respectively, underlining the relevance of this condition [6, 19]. Additionally, Nicely et al.. (2014) identified ASD in 13% and ADHD in 4% of ARFID cases [19]. Sanchez-Cerejo et al.. (2023) reported ASD prevalence in children with ARFID ranging from 8.2% to 54.75%. Heightened sensory sensitivity, a core feature of ASD, may contribute to food aversions commonly observed in ARFID [5].

In the present study, one of the five ARFID cases exhibited a specific aversion to cauliflower. Although we did not observe extreme aversions to entire food groups, this isolated observation aligns with reports of sensory-based avoidance, such as aversion to certain flavours or textures, that are frequently described in ARFID [12, 29]. In treatment-seeking cohorts, sensory sensitivity is the predominant presentation across age groups [30], and recent reviews note that most individuals with ARFID present sensory sensitivity or low appetite [31]. Given the very small size of our sample, we cite these studies solely to contextualise this phenomenon, acknowledging that our data cannot support any generalisations.

This study also explored the relationship between eating behaviour and ARFID. Higher mean scores ​​were observed in the FF and SR subscales, reflecting food fussiness and heightened satiety responsiveness—traits indicating low interest in food [23]. These findings align with Calisan Kinter et al.. (2024), who found significantly higher SR scores in children with ARFID and ASD compared to both ASD-only and typically developing children (TDC) (all *p* < 0.001) [32]. Similarly, Dovey et al. (2019) reported higher FF scores in children with ARFID and ASD compared to TDC (*p* < 0.001) [33].

Low scores were observed in the DD subscale, which is typically associated with sugary drink consumption and higher BMI [20] —not evident in the ARFID cases of this study. Further research is needed to clarify the impact of ARFID on children’s eating behaviours. Children self-regulate intake while caregivers decide what, when, and how food is offered. In ARFID cases, this autonomy may increase parental anxiety about nutritional adequacy [34].

Literature on food neophobia suggests that parental eating practices play a significant role, with maternal neophobic attitudes linked to higher levels of food neophobia in children. In response to feeding difficulties, parents may adopt strategies they perceive as effective, although some—such as using distractions like television or toys during meals—can be counterproductive [16, 35].

Given the influence of parental attitudes on children’s diet, parental practices were assessed using the CFQ. The highest mean scores ​​were in the RESP and MN dimensions, and the lowest were in the CN and PCW. Notably, PE was more pronounced in children with ARFID. These findings are consistent with Kozak et al.. (2023), who observed higher PE scores in children with ASD and ARFID compared to non-clinical samples [18]. Several studies reinforce the impact of parental practices on children’s eating behaviours [18, 24]. However, as this is a cross-sectional study, no causal inferences can be made.

This study presents limitations that may have influenced the results. Children attending this private paediatric nutrition clinic were not referred for suspected eating disorders; they came for routine consultations, which may have led to an under‑estimation of ARFID cases. In addition, the private setting means the sample may not reflect the sociodemographic or clinical diversity of public healthcare populations, as families with lower socioeconomic status may be underrepresented. Future research would benefit from larger, randomised samples to improve representativeness and strengthen the validity of results.

The fact that it is a cross-sectional study limits the results, as it presents the prevalence and characteristics of participants at a given moment, not allowing for the establishment of causal relationships or the monitoring of their development over time. Longitudinal studies would better identify risk factors and developmental trajectories.

Although paediatricians assessed ARFID cases, the involvement of child psychiatry or psychology would have improved diagnostic accuracy and increased case identification.

The transition from DSM-5 to DSM-5-TR may have influenced prevalence estimates in reference studies, affecting comparability. Additionally, the limited number of complete parental responses among ARFID cases restricted the ability to analyse associations with sex, age, BMI, or parental practices, highlighting the need for studies with larger, more comprehensive samples.

## Conclusion

This study identified a 3.1% prevalence of ARFID in Portuguese children aged 2–10 years, with higher rates in younger males. Raising awareness among parents, caregivers, and healthcare professionals is crucial for enhancing knowledge and improving the management of this disorder. Further research is needed to improve diagnostic tools and develop individualised, effective treatment strategies that promote healthy eating behaviours and ensure appropriate growth and development in affected children.

## Data Availability

The datasets used and/or analysed during the current study are available from the corresponding author upon reasonable request.

## Abbreviations

ARFID: Avoidant/Restrictive Food Intake Disorder
AN: Anorexia Nervosa
BN: Bulimia Nervosa
DSM-5: Diagnostic and Statistical Manual of Mental Disorders − 5^TH^ Edition
FED: Feeding and Eating Disorders
DSM-5-TR: 5^TH^ Edition Diagnostic and Statistical Manual of Mental Disorders − 5^TH^ Edition, Text Revision
UK: United Kingdom
AD: Anxiety disorder
ASD: Autism spectrum disorder
ADHD: Attention-Deficit/Hyperactivity Disorder
CEBQ: Children’s Eating Behaviour Questionnaire
SR: Satiety responsiveness
SE: Slowness in eating
FF: Food fussiness
EUE: Emotional undereating
FR: Food responsiveness
EF: Enjoyment of food
EOE: Emotional overeating
DD: Desire to drink
CFQ: Child Feeding Questionnaire
RESP: Perceived responsibility
PPW: Parent perceived weight
PCW: Perceived child weight
CN: Parents’ concerns about child weight
RST: Restriction
PE: Pressure to eat
MN: Monitoring
BMI: Body Mass Index
